# ABCG2 contributes to the development of gout and hyperuricemia in a genome-wide association study

**DOI:** 10.1038/s41598-018-21425-7

**Published:** 2018-02-16

**Authors:** Chung-Jen Chen, Chia-Chun Tseng, Jeng-Hsien Yen, Jan-Gowth Chang, Wen-Cheng Chou, Hou-Wei Chu, Shun-Jen Chang, Wei-Ting Liao

**Affiliations:** 1 Division of General Internal Medicine, Department of Internal Medicine, Kaohsiung Medical University Hospital, Kaohsiung, Taiwan; 2 Department of Internal Medicine, College of Medicine, Kaohsiung Medical University, Kaohsiung, Taiwan; 3 Department of Internal Medicine, Kaohsiung Municipal Ta-Tung Hospital, Kaohsiung, Kaohsiung Medical University, Kaohsiung, Taiwan; 4 Division of Rheumatology, Department of Internal Medicine, Kaohsiung Medical University Hospital, Kaohsiung, Taiwan; 5 Graduate Institute of Medicine, College of Medicine, Kaohsiung Medical University, Kaohsiung, Taiwan; 6 Department of Laboratory Medicine and Epigenome Research Center, China Medical University Hospital, China Medical University, Kaohsiung, Taichung Taiwan; 7 Institute of Biomedical Sciences, Academia Sinica, Taipei, Taiwan; 8 Department of Kinesiology, Health and Leisure Studies, National University of Kaohsiung, Kaohsiung, Taiwan; 9 Department of Medical Research, Kaohsiung Medical University Hospital, Kaohsiung, Taiwan; 10 Department of Biotechnology, College of Life Science, Kaohsiung Medical University, Kaohsiung, Taiwan

## Abstract

Although many genome-wide association studies (GWASs) of hyperuricemia or gout have been reported, the related genetic factors and the mechanisms from hyperuricemia to gouty attack remain unclear. This study aimed to identify genetic factors and pathogenesis of gout from hyperuricemia by genome-wide association study (GWAS). 747 gout patients, 747 hyperuricemia and 2071 age-matched controls were recruited and analyzed with Affymetrix 650 K chip to find the related genetic variants. The functions of the related genes were investigated in an endothelial cell (EC) with urate crystal stimulation. The GWAS results showed 36 SNPs to be strongly associated with gout compared to controls (all p-values < 10^−7^). Whereas the rs2231142 in ABCG2 gene had significant associations between gout and controls, between gout and hyperuricemia, and between hyperuricemia and controls (all p-values < 10^−7^), and the ORs were 4.34, 3.37 and 2.15 (all p-values < 0.001) after adjustment of potential confounders, respectively. The cell model showed significantly higher IL-8 release from EC combined with ABCG2 knockdown. We concluded that ABCG2 gene contributed to hyperuricemia but also gout, and that it was involved in the inflammation dysregulation via augmented IL-8 release in EC.

## Introduction

Gout is a common form of inflammatory arthritis characterized by recurrent attacks of acute inflammatory arthritis caused by monosodium urate (MSU) deposition and inflammation dysregulation. Since serum urate levels (SUA) and gout are heritable^1^, and most cases with hyperuricemia are asymptomatic, the genetic component of pathogenic mechanism remains unclear. Many genes are involved in urate transporter; SLC22A12^2^, SLC2A9^3,4^ and ABCG2^5–7^ have been reported to play important roles in the regulation of SUA, and their dysfunctions cause aberrant urate transport disorders leading to hyperuricemia. Hyperuricemia and gouty arthritis have different pathogenic mechanisms. A number of genes have been reported only to be associated with gouty inflammation or urate phagocytosis, but not to be related to hyperuricemia, such as tumor necrosis factor alpha (TNF-α), toll-like receptor II (TLR-2), NACHT, LRR and PYD domains-containing protein 3 (NLRP3) inflammasome and type 2 cyclic GMP-dependent protein kinase (cGKII)^8–11^. However, the related genes of pathogenic processes from hyperuricemia to gouty inflammation are also unclear.

Genome-wide association studies (GWASs) have explored many genes associated with serum uric acid levels or gout, for instance, SLC2A9, SLC2A12, SLC22A12^12,13^ for urate transporter, and ABCG2, SLC2A9, BCAS3, RFX3, KCNQ1, SLC22A12 and SLC17A1 for gout disease with individuals of Asian or European descent^14–18^. However, common variants identified by GWASs associated both with serum urate levels and gout were reported only in individuals of European ancestry^19–21^ and in a Japanese population^14^. Because most gout-related genes were also associated with hyperuricemia, in the present study we intended to identify the risk loci related to gout from the status of hyperuricemia, and then investigated their function in a cell model.

## Results

In our study, 747 gout patients, 747 male individuals with hyperuricemia and 2071 male normal controls were included from the Taiwan biobank database which provided gout history, uric acid, creatinine, biochemical markers, demographic and Affymetrix TWB 650 K SNP chip data. The mean ages of gout patients, hyperuricemia patients and controls were 50.29 years (±10.49), 49.19 years (±10.73) and 49.72 years (±11.11), respectively, which did not show any significant difference (p = 0.149; Table 1). Uric acid, creatinine, triglycerides, fasting sugar, total cholesterol, high-density lipoprotein cholesterol (HDL-c), low-density lipoprotein cholesterol (LDL-c), body mass index (BMI), body fat rate, GOT and GPT showed higher significant differences among these three groups (all p-values < 0.010; Table 1). The aforementioned variables were then taken as potential confounders for further analysis. For the GWAS analysis, the results showed that 36 SNPs located on chromosome 4 were significantly associated with gout disease compared to normal control (Supplementary Table 1), and one SNP (polymorphism rs2231142) showed significant associations between gout and hyperuricemia, and between hyperuricemia and normal controls (all p-values < 1 × 10^−7^). As known that data overfitting is always a challenge in biomarker discovery^22^, therefore we did a re-sampling to perform a non-matched design to analysis the related gene markers for gout. The result showed a total of 37 SNPs were related to gout while compared to normal controls (Supplementary Figure 1), ie, there were 36 SNPs between these two designs showed the same significant association (p < 10^−**7**^). All these significant SNPs passed 1% of false discovery rate (FDR) for association with gout or hyperuricemia.

**Table 1:** Demographic and biochemical data among all participants.

	Gout^a^	Hyperuricemia^b^	Normal controls^c^	p-values, comparisons
No.	747	747	2071	
Age (years; mean ± SD)				
	50.29 ± 10.49	49.19 ± 10.74	49.72 ± 11.11	0.149
Uric acid (mg/dl; mean ± SD)				
	7.72 ± 1.70	7.82 ± 0.82	5.71 ± 0.83	<0.001; a > c, b > c
Creatinine (mg/dl; mean ± SD)				
	1.05 ± 0.99	0.96 ± 0.54	0.87 ± 0.31	<0.001; a > b, b > c, a > c
BMI (kg/m^2^; mean ± SD)				
	26.52 ± 3.62	26.34 ± 3.40	24.46 ± 3.18	<0.001; a > c, b > c
Body fat rate (%; mean ± SD)				
	24.80 ± 5.65	24.88 ± 5.23	21.98 ± 5.30	<0.001; a > c, b > c
Diabetes (%)				
	74 (9.91)	39 (5.22)	151 (7.29)	0.002;
Sugar (fasting; mg/dl; mean ± SD)				
	102.98 ± 30.74	98.49 ± 16.52	100.17 ± 25.83	0.002; a > c, a > b
Total cholesterol (mg/dl; mean ± SD)				
	194.95 ± 34.36	200.14 ± 43.25	188.50 ± 34.10	<0.001; a > c, b > c, b > a
HDL (mg/dl; mean ± SD)				
	45.86 ± 10.59	45.73 ± 10.25	49.34 ± 10.97	<0.001; a < c, b < c
LDL (mg/dl; mean ± SD)				
	123.06 ± 30.68	127.60 ± 34.93	120.87 ± 30.61	<0.001; a < b, b > c
Triglycerides (mg/dl; mean ± SD)				
	163.95 ± 120.58	170.48 ± 169.22	117.17 ± 71.86	<0.001; a > c, b > c
SGOT (mg/dl; mean ± SD)				
	28.55 ± 13.62	28.28 ± 13.71	25.30 ± 12.10	<0.001; a > c, b > c
SGPT (mg/dl; mean ± SD)				
	33.38 ± 23.91	34.88 ± 26.11	27.09 ± 20.72	<0.001; a > c, b > c

The p-values were estimated by one-way analysis of variance and chi-square test, and posterior comparison was estimated by Bonferroni correction (significance for p < 0.017). Since the distributions of total cholesterol, triglycerides, high-density lipoprotein cholesterol (HDL-c), low-density lipoprotein cholesterol (LDL-c) and fasting glucose were far away from normally distribution, so the p-values were estimated by Kruskal-Wallis test. SGOT: serum glutamic oxaloacetic transaminase; SGPT: serum glutamic pyruvic transaminase; BMI: body mass index.

**Figure 1:**
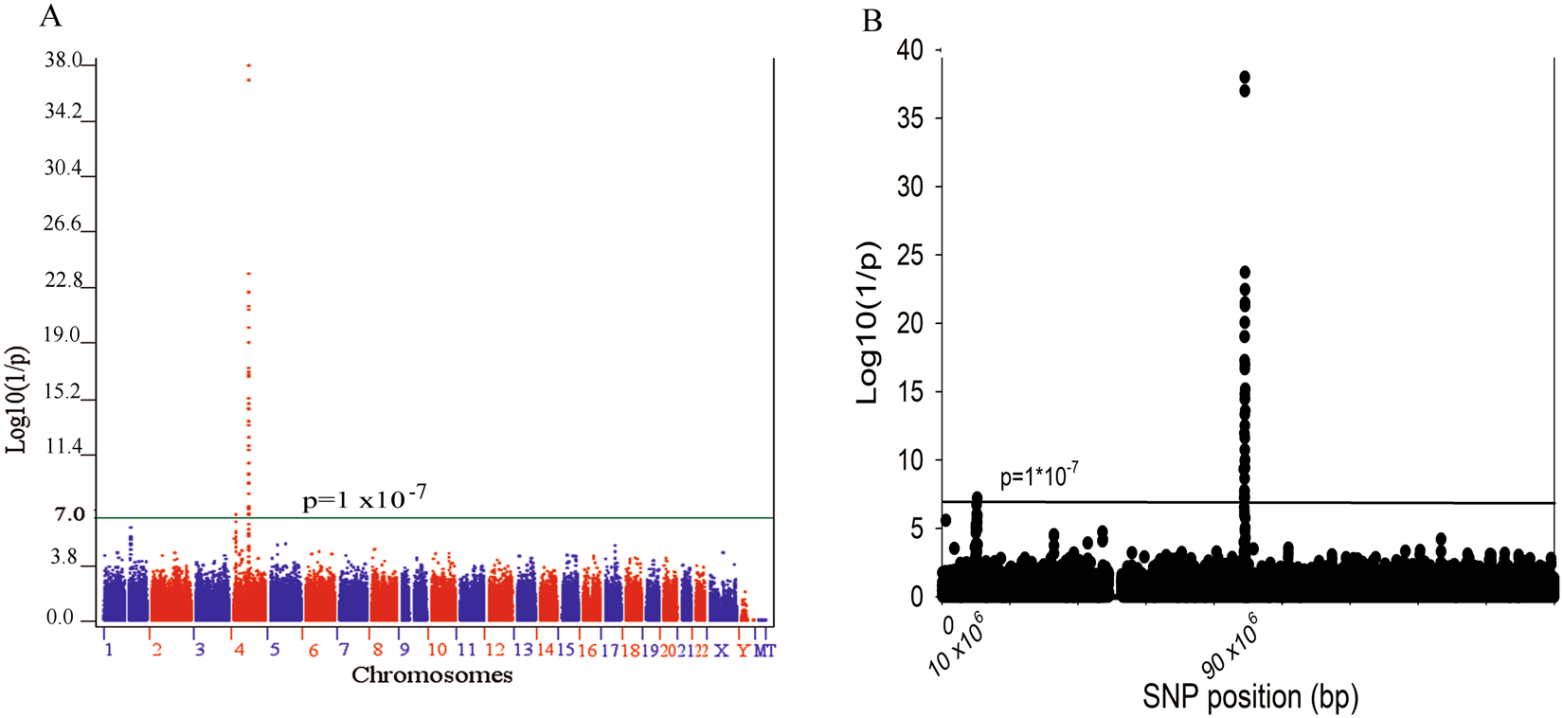
The Manhattan plot displays p-values of SNPs from 23 pairs of chromosomes and mitochondria and shows the genes on chromosome 4 were the hot spot for gout-susceptible genes (**A**). The log (1/p) of SNPs associated with gout compared to normal control in chromosome 4 (**B**). It showed two spots positioned near in 1 × 10^7^ (SLC2A9 gene) and in region of 8 × 10^7^ and 9 × 10^7^ near or in MEPE, SPP1 PKD2 and ABCG2 genes involved in gout occurrence.

The Manhattan plot displayed all p-values of SNPs across 23 pairs and mitochondria chromosomes and showed the genes on chromosome 4 were the hot spot for gout-susceptible genes (Fig. 1A). Figure 1B shows the p-values after negative logarithm transformation for the SNPs of gout compared to normal control in chromosome 4. It shows two spots positioned near in the region of 1 × 10^7^ base-pair and in the region between 8 × 10^7^ and 9 × 10^7^ bases. The latter region revealed that ABCG2 gene was the major gene contributing to gout disease, and MEPE, SPP1, and PKD2 were also involved. However, the first region consisted of four non-redundant genes encoded around rs9999470 within one mega-bases (1 Mb) and the expression quantitative trait loci (eQTL) analysis revealed higher association between the expressions of SLC2A9 and polymorphism rs9999470, but not WDR1, ZNF518B or CLNK genes (Supplementary Figure 2).

**Figure 2:**
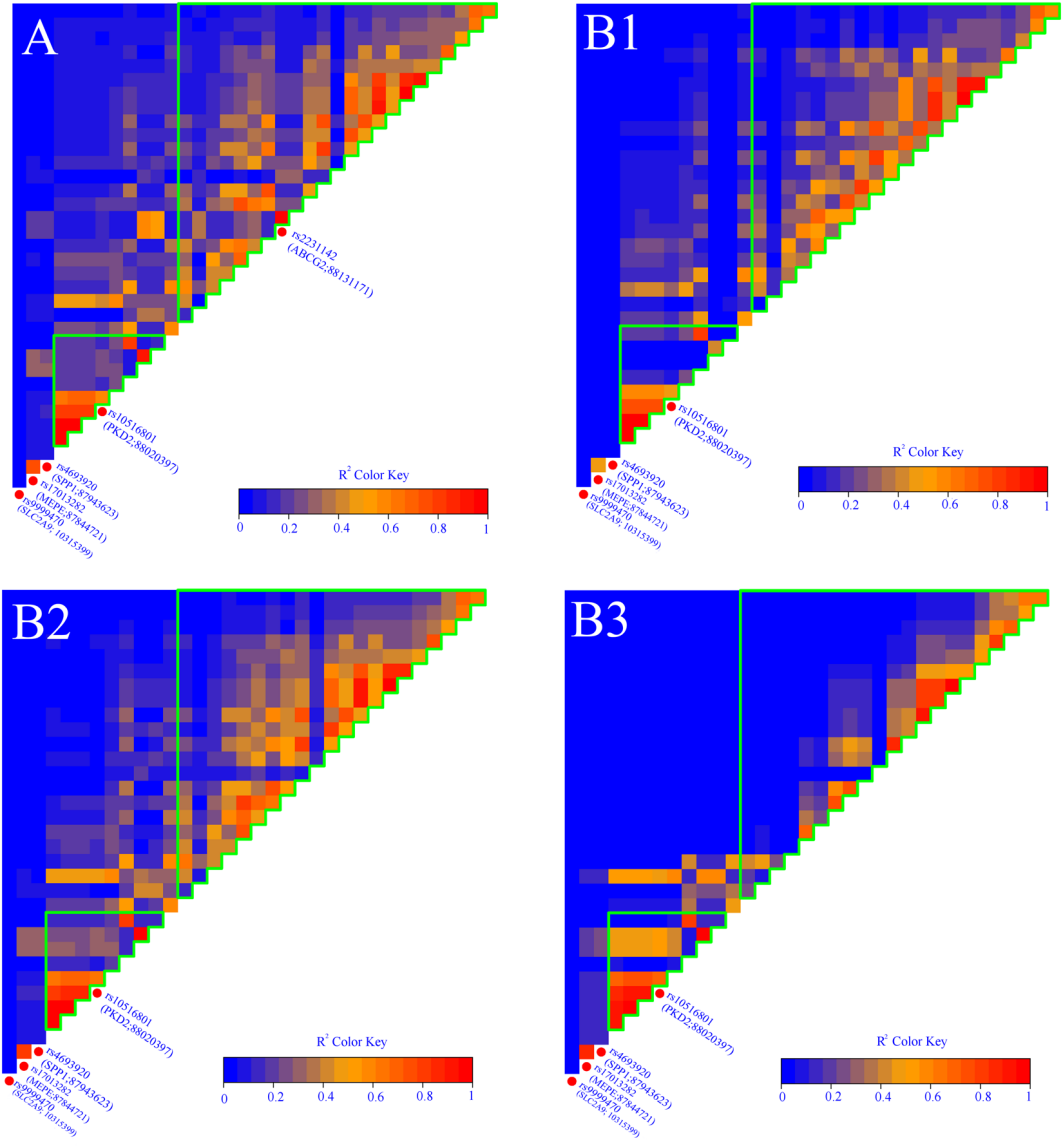
The linkage disequilibrium (LD) maps. Figure **A** is the result of comparing gout to normal control ignoring rs2231142 genotype, while Figure **B1**, **B2** and **B3** displays the same LD maps with different rs2231142 genotypes, namely GG, GT, and TT, respectively. Parenthesis indicates the gene name and position. Green line blocks the SNPs of the same genes.

A linkage disequilibrium test was further performed to eliminate redundant SNPs with genetic associations during genetic recombination (Fig. 2). Since the polymorphism rs2231142 in ABCG2 gene showed most significant association with gout or hyperuricemia, we displayed the LD according to different rs2231142 genotypes. Figure 2A shows the LD association between gout and normal control ignoring rs2231142 genotypes, while Fig. 2-B1, B2 and B3 display the same LD maps with different rs2231142 genotypes in GG, GT and TT, respectively.

Concerning the effect of polymorphism rs2231142 with different genotypes on the occurrence of gout disease from hyperuricemia, the OR was 1.84 (95% C I = 1.45–2.34) in genotype GT of rs2231142, and increased to 3.37 in genotype TT as compared to genotype GG after adjustment of potential confounders (Table 2). The same phenomenon of increased OR also was observed in comparisons of between gout and controls as well as between hyperuricemia and controls (Table 2). Moreover, the polymorphism rs9999470 showed weak association effect for the developments of gout and hyperuricemia after adjustment of potential confounders (Table 2).

**Table 2:** Associations of gout/hyperuricemia with control in different rs2231142 genotypes.

	Gout n (%)	Hyperuricemia n (%)	Control n (%)	OR1 (95% CI)	OR2 (95% CI)	OR3 (95% CI)
rs2231142 (ABCG2)						
GG	193 (25.84)	304 (40.75)	1088 (52.56)	1.0	1.0	1.0
GT	385 (51.54)	352 (47.18)	812 (39.23)	2.37 (1.81–3.09)***	1.84 (1.45–2.34)***	1.67 (1.37–2.03)***
TT	169 (22.62)	90 (12.06)	170 (8.21)	4.34 (2.97–6.36)***	3.37 (2.43–4.68)***	2.15 (1.57–2.94)***
rs9999470 (SLC2A9)						
TT	322 (43.11)	377 (50.47)	1126 (54.48)	1.0	1.0	1.0
CT	346 (46.32)	304 (40.70)	808 (39.09)	1.20 (0.93–1.54)	1.36 (1.09–1.70)**	1.11 (0.91–1.34)
CC	79 (10.58)	66 (8.84)	133 (6.43)	1.65 (1.07–2.54)*	1.41 (0.98–2.05)	1.50 (1.07–2.12)*

OR1: Odds ratio of gout compared to control. OR2: Odds ratio of gout compared to hyperuricemia. OR3: Odds ratio of hyperuricemia compared to control. OR: Odds ratios; 95% CI: 95% confidence intervals. OR1 and OR2 were adjusted by age, uric acid, creatinine, BMI, body fat rate, total cholesterol, triglycerides, high-density lipoprotein cholesterol, low-density lipoprotein cholesterol, serum glutamic oxaloacetic transaminase, serum glutamic pyruvic transaminase and fasting glucose; OR3 was adjusted by the same variables as OR1 but not uric acid. *p-value < 0.05; **p-value < 0.01; ***p-value < 0.001.

Furthermore, since polymorphism rs2231142 provided the highest association with development of gout disease, we performed another analysis for polymorphism rs2231142 with representative SNPs of the other four genes (Table 3). The polymorphism rs9999470 showed a significant association with polymorphism rs2231142 on gout occurrence; it showed that those with rs9999470 genotypes CT and CC interacted with rs2231142 genotype GT to increase gout risk in a dose-dependent manner. ORs increased from 2.07 to 2.67 and 4.94 in genotypes TT, CT and CC of rs9999470 among those with rs2231142 genotype GT after adjusting potential confounders, respectively (all p- values < 0.001; Table 3). Moreover, in those with rs2231142 genotype TT, the associated effect after adjusting potential confounders was clearer; the ORs increased from 4.24 to 4.64 and 21.32 (all p- values < 0.001; Table 3). An additive interaction effect was observed in those combined with genotype CC of rs9999470 and genotypes GT and TT of rs2231142 (p < 0.05; Table 3). This implied that polymorphism rs9999470 acted as an additive associated factor for developing gout, which was dependent on the genotypes of polymorphism rs2231142.

**Table 3:** Associations of gout occurrence compared to normal controls between polymorphism rs2231142 and polymorphisms rs9999470, rs17013282, rs4693920 and rs10516801.

SNP (Gene)	rs2231142 (ABCG2) OR (95% CI) gout/control		
	GG (n = 1585)	GT (n = 1549)	TT (n = 429)
rs9999470 (SLC2A9)			
TT	1.0 86/584	2.07 (1.41–3.05)*** 161/453	4.24 (2.41–7.47)*** 75/89
CT	1.17 (0.75–1.81) 92/426	2.67 (1.78–4.02)*** 179/308	4.64 (2.59–8.34)*** 75/73
CC	0.85 (0.37–1.97) 15/77	4.94 (2.53–9.67)***^§^ 45/49	21.32 (5.11–88.99)***^§^ 19/7
rs17013282 (MEPE)			
GG	1.0 175/1254	2.61 (1.88–3.63)*** 218/688	4.33 (2.40–7.83)*** 71/105
GA	0.93 (0.44–1.97) 18/135	2.18 (1.54–3.09)*** 159/458	4.48 (2.67–7.52)*** 75/120
AA	−0/0	6.37 (1.68–24.09)** 8/17	3.77 (1.47–9.67) ** 23/35
rs4693920 (SPP1)			
TT	1.0 165/1190	2.95 (2.10–4.14)*** 208/619	5.29 (2.89–9.68)*** 65/94
TC	1.07 (0.59–1.96) 26/194	1.98 (1.40–2.81)*** 161/508	3.97 (2.34–6.74)*** 74/125
CC	0.47 (0.01–19.67) 1/7	5.23 (2.08–13.13)*** 16/35	5.01 (2.13–11.78)*** 30/40
rs10516801 (PKD2)			
CC	1.0 37/206	2.69 (1.44–5.06)** 136/363	3.28 (1.64–6.57)*** 99/155
CT	1.02 (0.56–1.86) 103/671	1.70 (0.96–3.02) 206/654	5.17 (2.20–12.13)*** 60/91
TT	0.53 (0.27–1.06) 52/513	2.24 (1.06–4.73)* 43/146	9.95 (1.36–72.66)* 10/14

^*^p < 0.05; **p < 0.01; ***p < 0.001. SLC2A9: Solute carrier family 2, facilitated glucose transporter member 9; MEPE: Matrix, Extracellular, Phosphoglycoprotein; SPP1: Secreted Phosphoprotein 1; PKD2: Polycystin 2; ABCG2: ATP-Binding Cassette, Subfamily G, Member 2; OR: odds ratio; 95%CI: 95% confidence intervals. OR was adjusted by age, uric acid, creatinine, BMI, body fat rate, total cholesterol, triglycerides, high-density lipoprotein cholesterol, low-density lipoprotein cholesterol, serum glutamic oxaloacetic transaminase, serum glutamic pyruvic transaminase and fasting glucose; §: The p-value was estimated for additive interaction model (p < 0.05).

**Figure 3:**
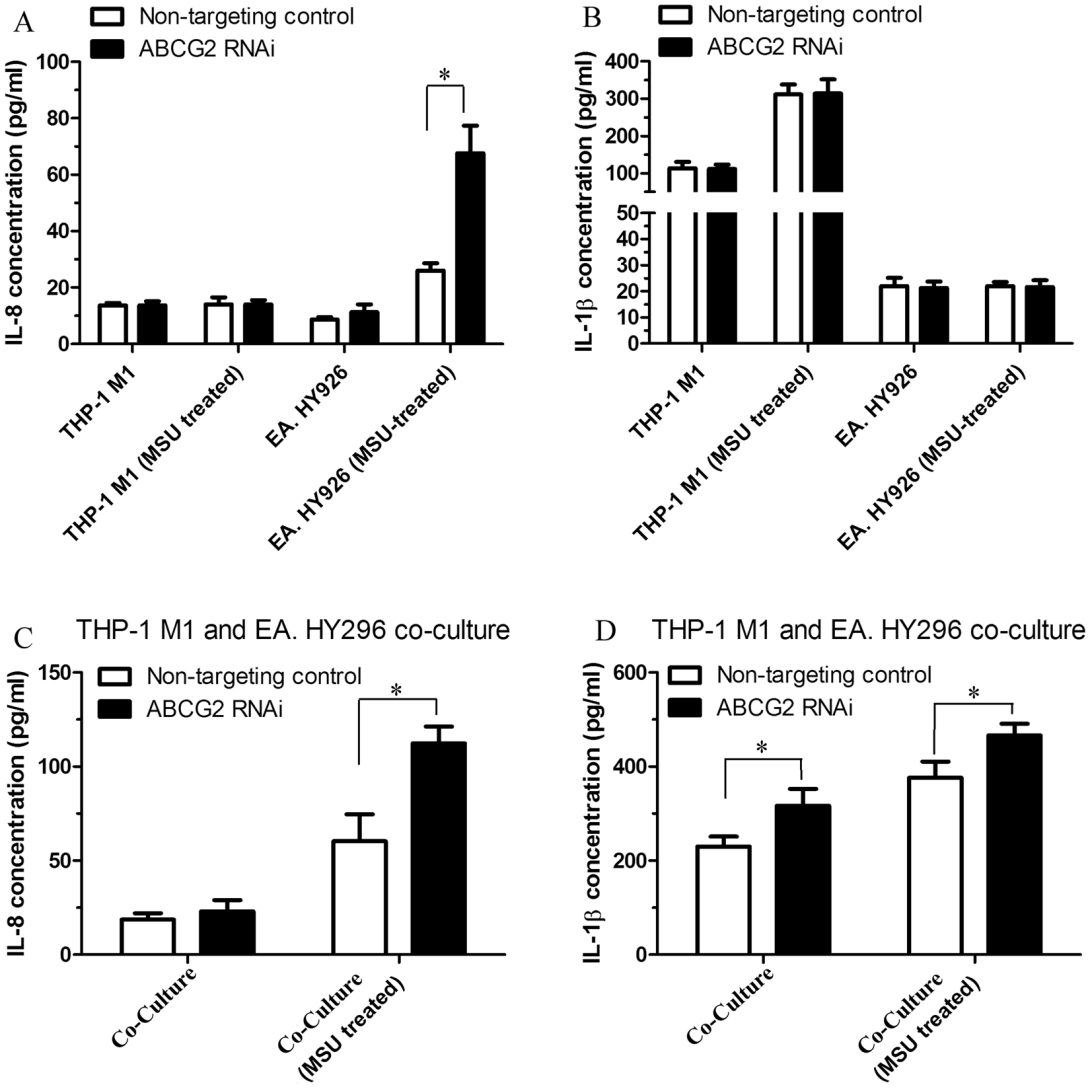
PMA-primed THP-1 and endothelial cell line EA. HY296 were used under conditions of with or without ABCG2 gene knockdown (ABCG2 RNAi). It showed IL-8 released was significantly higher from ABCG2 knockdown EA. HY296 cell after urate stimulation (p < 0.05), but not in PMA-primed THP-1 cells (p > 0.05) (**A**). The IL-1β release did not show significant increases either in THP-1 or EA. HY296 cells even under the condition of ABCG2 knockdown or urate stimulation (both p-values > 0.05) (**B**). A co-culture of THP-1 and EA. HY296 showed the releases of both IL-8 and IL-1β were significantly increased after urate stimulation and ABCG2 knockdown (both p-values < 0.05) (**C** and **D**). *p < 0.05.

Our study found that ABCG2 contributed not only to hyperuricemia but also to gouty inflammation. Previously, some studies demonstrated that ABCG2 contributed to hyperuricemia, which was caused by renal urate overload and intestinal urate under-excretion^23–26^. In addition, ABCG2 knockout mice showed increased inflammatory responses, including increased IL-8 release in the brain via regulating NF-κB activation^27^. Therefore, we performed cell study to validate ABCG2-associated inflammatory responses instead of urate excretion, IL-1β and IL-8 cytokines release with MSU crystals stimulation. PMA-primed THP-1 (under a macrophage M1-like inflammatory stage) and EC line EA. HY296 were used with and without ABCG2 gene knockdown (ABCG2 RNAi). The results showed that IL-8 release was significantly higher from ABCG2 knockdown EA. HY296 cell after urate crystal stimulation (p < 0.05), but this was not observed in PMA-primed THP-1 cells (Fig. 3A). Meanwhile, the IL-1β release did not show significantly increases in either THP-1 or EA. HY296 cells even under the condition of ABCG2 knockdown or urate crystal stimulation (both p-values > 0.05, Fig. 3B). These data suggested that ABCG2 dysfunction (knockdown) promoted MSU-induced IL-8 release from EC, but not in M1-like macrophages. Furthermore, with a combination of THP-1 and EA. HY296, the results showed the releases of both IL-8 and IL-1β were significantly increased after urate crystal stimulation and ABCG2 knockdown (both p-values < 0.05, Fig. 3C,D). These results implied that, under conditions of ABCG2 knockdown and MSU crystals-stimulation, the release of IL-8 from EC (EA. HY296) was able to enhance inflammatory cytokine IL-1β release with the involvement of macrophages (THP-1). Overall, the cell model showed that ABCG2 knockdown increased the MSU crystals-induced inflammation responses through IL-8 release in EC.

## Discussion

This study demonstrates that ABCG2 gene contributes to the development of not only hyperuricemia but also gout with solid evidence from the GWAS and endothelial cell model. Moreover, this might be the first research to clarify the mechanism of ABCG2 leading to gouty inflammation involving the release of IL-8 upon MSU crystals-stimulation in EC. Previously, many studies showed that ABCG2 played an important role in renal urate overload and extra-renal urate under-excretion, especially intestinal excretion^23,24,28–30^. However, the development of gout needs both hyperuricemia and aberrant inflammatory mechanism, possibly involving IL-1β, TNF-α and IL-8, etc. In our knowledge, few previous studies determined whether ABCG2 gene dysfunction could be associated with aberrant production of inflammatory cytokines in gout. Shen *et al*. showed that ABCG2 gene could relieve oxidative stress and inflammatory response via inhibiting NF-κB signaling pathway in cell models and brain^27^, and recently oxidative stress has proved to be a key for gouty arthropathy^31,32^. Our current study showed that ABCG2 knockdown induced increase of IL-8 release after urate crystal stimulation. This finding suggested that ABCG2 dysfunction contributed to gouty inflammation.

The mechanism causing acute inflammation in gout patients initiates from MSU crystal deposition, and then MSU interacts with macrophages to activate NLRP3 inflammasome and IL-1β release. Once IL-1β is released, it orchestrates a series of events leading to EC activation and neutrophils recruitment. Upon EC activation, several cytokines are released, particularly IL-8, which is the key for neutrophils recruitment. Nishimura *et al*. provided evidence that IL-8 release was related to MSU crystal-induced gouty arthritis^33^. Gagne *et al*. demonstrated that reduced myeloid inhibitory C-type lectin (MICL) expression was associated with augmented inflammatory responses from neutrophils, and that a higher level of neutrophil MICL expression was associated with reduced IL-8 production^34,35^. Therefore, the molecular basis for IL-8 regulation in EC may be the key to clarifying the mechanisms of gout flare. Here, we propose that ABCG2 gene may play an important role in release of IL-8 for further neutrophilic recruitment. Therefore, in case of ABCG2 dysfunction, the subjects have hyperuricemia first by renal urate overload and intestinal urate under-excretion; and then increased release of IL-1β (by macrophages) and IL-8 (by ECs) invoke further neutrophilic recruitment and gouty flare.

The polymorphism rs2231142 in ABCG2 gene, which causes a Glu141Lys amino acid substitution, accounted for 0.57% of the variation in serum urate^36^, and a functional study of the rs2231142 has shown that it causes a 53% reduction in the rate of ABCG2-mediated urate transport compared with wild-type^5,37^. Even though polymorphism rs2231142 acted as a risk factor for gout development, our study also found that polymorphism in SLC2A9 gene revealed associated effect dependent on rs2231142 genotypes. For example, polymorphisms rs9999470 which regulates SLC2A9 function showed an additive significant association with gout in those with polymorphism rs2231142 genotype TT. SLC2A9 coding for the glucose-facilitated transporter GLUT9^36^, some studies showed renal hypouricemia was caused by dysfunction in the SLC2A9 gene via its decreased urate reabsorption on the renal proximal tubules^3,4^. Here, our study demonstrates that those with mutants in both ABCG2 and SLC2A9 genes cause an additive interaction effect for gout occurrence.

This study also explores whether PKD2 genes may interact with rs2231142 genotypes in the development of gout. Polymorphism rs10516801 in PKD2 gene showed risk effects in those with rs2231142 genotypes GT or TT. PKD2 gene encodes a member of the polycystin protein family; the protein is an integral membrane protein involved in cell-cell/matrix interactions, which may function in renal tubular development, morphology and function, and modulate intracellular calcium homoeostasis and other signal transduction pathways^38^. Lee *et al*. applied pathway analysis to GWAS data on uric acid levels and found PKD2 contributed to uric acid levels by affecting ion transmembrane transporter activity^39^. According to the Genecards, PKD2 is not only expressed in kidney but also in white blood cells, especially in B cells. Surprisingly, the role of B lymphocytes related to gout development has only recently been recognized^40,41^, it may be involved in the gouty inflammation mediated by B cells.

In conclusion, based upon GWAS of gout and hyperuricemia simultaneously, we revealed that ABCG2 gene contributed to the development of not only hyperuricemia but also gout, affecting the IL-8 release from EC to cause dysregulation of inflammation and the development of gouty attack.

## Methods

### Study population

Our study aimed to provide risk genome components of developing gout and hyperuricemia based on GWAS. We recruited 747 male gout patients, 747 male individuals with hyperuricemia (uric acid levels > = 7.0 mg/dl; without gout disease history) and 2071 male normal controls (with neither gout nor hyperuricemia) from the Taiwan biobank database which provided gout history, uric acid, creatinine, biochemical, demographic and Affymetrix TWB 650 K SNP chip data.

The cohort was selected based on community population initiated by Academia Sinica in Taiwan from 2012, which collect the DNA specimen of a large group to understand the relationships between genetics, environmental exposure and the etiology/progression of disease, including gout, diabetes, and hypertension. Enrollees of the Taiwan biobank have undergone physical examination, and provided blood and urine samples for biochemical test and detailed health information about their lifestyles and gout disease history for further analysis. Gout status was obtained by self-report, which has been evidenced to be the best test performance characteristics of existing definitions with sensitivity 80% and specificity 72%^42^. This research project was approved by the ethics committee of Antai-Tian-Sheng memorial Hospital Institutional Review Board (TSMHIRB 16-006-C0). The study was conducted in accordance with the principles of the Declaration of Helsinki and the Good Clinical Practice Guidelines, and all the participants were informed consent.

### SNP genotyping and quality control

Blood DNA samples from Taiwan biobank participants were genotyped using TWB 650 K SNPs array designed by Affymetrix (Affymetrix, Santa Clara, California, USA) for selected SNPs^43^. For TWB SNPs array, we used the Affymetrix Power Tools (APT) and performed a standard quality control procedure to exclude SNPs with low call rate (<99%), p value for the Hardy-Weinberg equilibrium test of <1.0 × 10^−4^ for controls and minor allele frequency of <0.01. In total, we obtained 537,478 SNPs for analysis in the screening stage.

### Cell culture

EA. HY296, an immortalized human endothelial cell (EC), was cultured in DMEM medium with 10% FBS at 37 °C in a humidified incubator with 5% CO_2_ atmosphere, with or without 7.0 mg/dl of monosodium urate crystals (MSU; Sigma, St. Louis, MO) treatment for 48 hours. THP-1, a human acute monocytic leukemia cell line, was cultured in RPMI 1640 medium with 10% FBS at 37 °C in a humidified incubator with 5% CO_2_ atmosphere. THP-1 monocyte has the potential to polarize into macrophage-like cells and functionally regulates purified T cells^44^. We treated THP-1 with 0.2 μg/ml of phorbol myristate acetate (PMA; Sigma, St. Louis, MO) for 3 days to activate it to a macrophage-like (M1-like) phenotype. The M1-like THP-1 cells were treated with 7.0 mg/dl of MSU crystals (Sigma, St. Louis, MO) for 48 hours.

### ABCG2 siRNA synthesis and transfection

Using the siGENOME SMARTpool system, human ABCG2 siRNA was chemically synthesized and one tube containing a mixture of SMART selection-designed siRNA targeting gene for *in-vitro* transfection was prepared. The siGENOME non-targeting siRNA pool was also performed in the same way (Thermo, Dharmacon, Inc. DBA). EA. HY296 or THP-1 cells were seeded into 24-well culture plates and transfected with 100 nM of siRNA per well using Xfect^TM^ siRNA transfection reagent (Clontech, CA, USA), according to the manufacturer’s instruction. Cells were analyzed at 48 hours after transfection.

### ELISA tests for interleukin-8 (IL-8) and IL-1β

We selected IL-8 and IL-1β as detection targets based on the identified gouty inflammatory factors. After treatment, the cell-free supernatant from cultured EA. HY296 or THP-1 cells was obtained for IL-8 and IL-1β release measurement using commercially available ELISA kits (Quantikine, R&D System, Minneapolis, MN) according to the manufacturer’s instructions. IL-8 and IL-1β cytokine concentrations were calculated based on linear regression standard curves where r^2^ was higher than 0.99. The detection ranges were 1.6–1000 pg/ml for ELISA kits.

### Statistical and expression quantitative trait loci (eQTL) analysis

The associations of SNPs for gout or hyperuricemia occurrence in the GWAS were tested by chi-square tests, odds ratios (ORs) and 95% confident intervals (95% CI), and a Manhattan Plot was made to show the p-values across all the 22 pairs, x, y and mitochondria chromosomes. A false discovery rate (FDR) was applied for analysis of further association with gout, and a linkage-disequilibrium (LD) test was drawn using the R program (LDheatmap). A one-way analysis of variance (ANOVA) was applied to estimate the mean differences of demographic and biochemical data among those gout patients, hyperuricemia and controls. A logistic regression model was applied to estimate the associations between gout phenotype and genetic variants after adjusting potential variables, such as age, uric acid, creatinine, body mass index (BMI), body fat rate, total cholesterol, triglycerides, high-density lipoprotein cholesterol (HDL-c), low-density lipoprotein cholesterol (LDL-c), serum glutamic oxaloacetic transaminase (GOT), serum glutamic pyruvic transaminase (GPT) and fasting glucose. The genotypes associated with genes expression in whole-blood samples were retrieved from genotype-tissue expression (GTEx) Portal V6p (www.gtexportal.org; eQTL), and statistical significance was determined using the website’s algorithm^25^. Significance after Bonferroni correction for multiple testing was considered for p values less than 1 × 10^−7^ in the part of GWAS, and it was 0.05 in the analysis of SNPs with demographic data.

## Electronic supplementary material

Supplementary Table1 1

Supplementary Figure 1

Supplementary Figure 2
