# Supplementary material for: ABCG2 contributes to the development of gout and hyperuricemia in a genome-wide association study
Source: Sci Rep. 2018 Feb 16;8:3137. doi: 10.1038/s41598-018-21425-7 (PMC5816657; doi:10.1038/s41598-018-21425-7)
Supplement: Supplementary file 1 — Supplementary Table1 1 [file 41598_2018_21425_MOESM1_ESM.pdf]

Title: ABCG2 contributes to the development of gout and hyperuricemia in a genome-wide association study

Chung-Jen Chen[1,2], Chia-Chun Tseng[3], Jeng-Hsien Yen[4,5], Jan-Gowth Chang[6], Wen-Cheng Chou[7], Hou-Wei Chu[7], Shun-Jen Chang[8,*], Wei-Ting Liao[9,*]

[1]Division of General Internal Medicine, Department of Internal Medicine, Kaohsiung Medical University Hospital, Kaohsiung, Taiwan.
[2]Department of Internal Medicine, College of Medicine, Kaohsiung Medical University, Kaohsiung, Taiwan.
[3]Department of Internal Medicine, Kaohsiung Municipal Ta-Tung Hospital, Kaohsiung, Kaohsiung Medical University, Kaohsiung, Taiwan.
[4]Division of Rheumatology, Department of Internal Medicine, Kaohsiung Medical University Hospital, Kaohsiung, Taiwan.
[5]Graduate Institute of Medicine, College of Medicine, Kaohsiung Medical University, Kaohsiung, Taiwan.
[6]Department of Laboratory Medicine and Epigenome Research Center, China Medical University Hospital, China Medical University, Taichung, Taiwan.
[7]Institute of Biomedical Sciences, Academia Sinica, Taipei, Taiwan.
[8]Department of Kinesiology, Health and Leisure Studies, National University of Kaohsiung, Kaohsiung, Taiwan.
[9]Department of Biotechnology, College of Life Science, Kaohsiung Medical University, Kaohsiung, Taiwan.

*Corresponding author: Shun-Jen Chang, Department of Kinesiology, Health and Leisure Studies, National University of Kaohsiung, Kaohsiung, Taiwan. No. 700, Kaohsiung University Road, Nanzih District, Kaohsiung city, Taiwan. Phone: +886-7-5916679; Fax: +886-7-5919264; E-mail: changsj1104@gmail.com; or Wei-Ting Liao, Department of Biotechnology, College of Life Science, Kaohsiung Medical University, Kaohsiung, Taiwan. Phone: +886-7-3121101 ext 2791; Fax: +886-7-3125339; E-mail: wtliao@kmu.edu.tw.

Supplementary Table 1 Odds ratios of gout occurrence in 36 significant SNPs from GWAS.

No.	SNP (gene; physical position; Functional Consequence)	Gout (n=747)	Controls (n=2071)	aOR (95% CI)	$-\log_{10}(p)$[¥]	p-values[§]
1	rs9999470 (SLC2A9;10315399)					
	TT	322 (43.11)	1126 (54.48)	1.0		
	CT	346 (46.32)	808 (39.09)	1.20 (0.93-1.54)	5.15	0.157
	CC	79 (10.58)	133 (6.43)	1.65 (1.07-2.54)	5.74	0.024
2	rs17013282 (MEPE; 87844721; intron)					
	GG	464 (62.12)	1522 (73.63)	1.0		
	GA	252 (33.73)	513 (24.82)	1.48 (1.14-1.92)	6.54	0.003
	AA	31 (4.15)	32 (1.55)	2.76 (1.34-5.72)	5.62	0.006
3	rs4693920 (SPP1; 87943623; upstream)					
	TT	438 (58.71)	1416 (68.44)	1.0		
	TC	261 (34.99)	596 (28.81)	1.23 (0.95-1.59)	3.81	0.118
	CC	47 (6.30)	57 (2.75)	2.61 (1.45-4.72)	6.15	0.002
4	rs61566498 (PKD2; 88001816; intron)					
	CC	307 (41.15)	652 (31.53)	1.0		
	CT	348 (46.65)	996 (48.16)	0.75 (0.58- 0.98)	2.88	0.037
	TT	91 (12.20)	420 (20.31)	0.51 (0.35-0.73)	8.27	<0.001
5	rs72873439 (PKD2; 88004103; upstream)					
	AA	298 (39.89)	630 (30.51)	1.0		
	AG	356 (47.66)	1005 (48.67)	0.78 (0.60 -1.02)	2.71	0.068

	GG	93 (12.45)	430 (20.82)	0.52 (0.36-0.75)	8.46	<0.001
6	rs12511569 (PKD2; 88004415; upstream)					
	CC	297 (39.76)	628 (30.40)	1.0		
	TT	357 (47.79)	1006 (48.69)	0.78 (0.60-1.01)	2.68	0.063
	TT	93 (12.45)	432 (20.91)	0.51 (0.36 -0.74)	8.57	<0.001
7	rs10516801 (PKD2; 88020397; intron)					
	CC	272 (36.46)	521 (25.18)	1.0		
	CT	369 (49.46)	1028 (49.69)	0.72 (0.55-0.94)	4.02	0.017
	TT	105 (14.08)	520 (25.13)	0.45 (0.32-0.65)	12.88	<0.001
8	rs17013735 (PKD2; 88038593; intron)					
	AA	288 (38.66)	593 (28.69)	1.0		
	AG	362 (48.59)	1027 (49.69)	0.85 (0.65-1.10)	3.18	0.215
	GG	95 (12.75)	447 (21.63)	0.55 (0.38-0.79)	9.42	0.001
9	rs17013739 (PKD2; 88042103; intron)					
	GG	505 (67.69)	1164 (56.29)	1.0		
	AG	221 (29.62)	785 (37.96)	0.67 (0.52-0.87)	5.52	0.002
	AA	20 (2.68)	119 (5.75)	0.52 (0.29-0.95)	4.11	0.033
10	rs2725211 (PKD2; 88049223; intron)					
	CC	316 (42.30)	1239 (59.94)	1.0		
	CT	343 (45.92)	729 (35.27)	1.62 (1.26-2.09)	10.93	<0.001
	TT	88 (11.78)	99 (4.79)	3.07 (1.93-4.88)	15.56	<0.001
11	rs2728104 (PKD2; 88051854; intron)					
	TT	294 (39.52)	1169 (56.58)	1.0		
	TC	360 (48.39)	786 (38.04)	1.64 (1.28-2.11)	10.44	<0.001

	CC	90 (12.10)	111 (5.37)	2.76 (1.76-4.34)	14.16	<0.001
12	rs74901820 (PKD2; 88060385; intron)					
	GG	500 (67.11)	1139 (55.21)	1.0		
	AG	222 (29.80)	790 (38.29)	0.71 (0.55-0.91)	5.83	0.007
	AA	23 (3.09)	134 (6.50)	0.51 (0.29-0.90)	4.53	0.020
13	rs1448784 (ABCG2; 88091168; UTR 3 prime)					
	AA	429 (57.43)	953 (46.13)	1.0		
	AG	276 (36.95)	899 (43.51)	0.73 (0.57-0.94)	4.69	0.015
	GG	42 (5.62)	214 (10.36)	0.52 (0.33-0.83)	5.70	0.006
14	rs2231164 (ABCG2; 88094705; intron)					
	TT	113 (15.15)	612 (29.61)	1.0		
	TC	395 (52.95)	1035 (50.07)	1.72 (1.26-2.34)	9.30	<0.001
	CC	238 (31.90)	420 (20.32)	2.46 (1.73-3.49)	17.80	<0.001
15	rs2725263 (ABCG2; 88105276; intron)					
	CC	266 (35.66)	564 (27.23)	1.0		
	AC	368 (49.33)	1011 (48.82)	0.87 (0.66-1.13)	2.16	0.294
	CC	112 (15.01)	496 (23.95)	0.50 (0.35-0.72)	8.18	<0.001
16	rs34472643 (ABCG2; 88108714; intron)					
	GG	483 (64.66)	1049 (50.75)	1.0		
	GA	244 (32.66)	852 (41.22)	0.67 (0.52-0.85)	6.78	0.001
	AA	20 (2.68)	166 (8.03)	0.34 (0.19-0.61)	8.39	<0.001
17	rs12505410 (ABCG2; 88109689; intron)					
	TT	414 (55.42)	863 (41.71)	1.0		
	TG	295 (39.49)	941 (45.48)	0.73 (0.57-0.94)	5.72	0.014

	GG	38 (5.09)	265 (12.91)	0.36 (0.23-0.59)	11.23	<0.001
18	rs2622621 (ABCG2; 88109768; intron)					
	GG	345 (46.25)	647 (31.42)	1.0		
	GC	323 (43.30)	996 (48.37)	0.69 (0.53-0.89)	7.18	0.005
	CC	78 (10.46)	416 (20.20)	0.39 (0.27-0.57)	13.89	<0.001
19	rs2231146 (ABCG2; 88118348; intron)					
	TT	518 (69.34)	1126 (54.45)	1.0		
	TC	218 (29.18)	812 (39.26)	0.65 (0.50-0.83)	8.25	<0.001
	CC	11 (1.47)	130 (6.29)	0.25 (0.12-0.52)	8.48	<0.001
20	rs2231142 (ABCG2; 88131171; missense)					
	GG	193 (25.84)	1088 (52.56)	1.0		
	GT	385 (51.54)	812 (39.23)	2.37 (1.81-3.09)	23.07	<0.001
	TT	169 (22.62)	170 (8.21)	4.34 (2.97-6.36)	41.62	<0.001
21	rs4148155 (ABCG2; 88133515; intron)					
	AA	192 (25.74)	1087 (52.51)	1.0		
	AG	385 (51.61)	812 (39.23)	2.39 (1.83-3.12)	23.04	<0.001
	GG	169 (22.65)	171 (8.26)	4.38 (2.99-6.41)	41.62	<0.001
22	rs12641369 (ABCG2; 88138765; intron)					
	GG	412 (55.38)	867 (41.88)	1.0		
	GA	284 (38.17)	930 (44.93)	0.69 (0.54-0.89)	6.03	0.004
	AA	48 (6.45)	273 (13.19)	0.45 (0.29-0.70)	9.00	<0.001
23	rs77377473 (ABCG2; 88140650; intron)					
	AA	569 (76.48)	1238 (60.18)	1.0		
	AG	166 (22.31)	723 (35.15)	0.58 (0.44-0.76)	11.67	<0.001

	GG	9 (1.21)	96 (4.67)	0.27 (0.12-0.61)	6.18	0.002
24	rs2725252 (ABCG2; 88140758; intron)					
	CC	358 (48.12)	734 (35.51)	1.0		
	CA	317 (42.61)	989 (47.85)	0.76 (0.59-0.98)	5.41	0.031
	AA	69 (9.27)	344 (16.64)	0.47 (0.32-0.69)	8.06	<0.001
25	rs3114018 (ABCG2; 88143429; intron)					
	CC	407 (54.70)	738 (35.69)	1.0		
	CA	282 (37.90)	991 (47.92)	0.57 (0.45-0.74)	12.49	<0.001
	AA	55 (7.39)	339 (16.39)	0.35 (0.23-0.53)	15.13	<0.001
26	rs3109823 (ABCG2; 88143450; intron)					
	TT	551 (73.76)	1178 (56.94)	1.0		
	TC	188 (25.17)	763 (36.88)	0.62 (0.44-0.81)	10.70	<0.001
	CC	8 (1.07)	128 (6.19)	0.22 (0.10-0.49)	9.72	<0.001
27	rs2725244 (ABCG2; 88147609; intron)					
	CC	435 (58.47)	784 (38.00)	1.0		
	CT	269 (36.16)	997 (48.33)	0.53 (0.41-0.68)	14.85	<0.001
	TT	40 (5.38)	282 (13.67)	0.30 (0.20-0.49)	15.05	<0.001
28	rs2622604 (ABCG2; 88157772; intron)					
	CC	558 (74.70)	1217 (58.76)	1.0		
	CT	181 (24.23)	755 (36.46)	0.57 (0.44-0.75)	9.00	<0.001
	TT	8 (1.07)	99 (4.78)	0.33 (0.15-0.73)	6.82	0.006
29	rs2622605 (ABCG2; 88158234; intron)					
	CC	429 (57.82)	786 (37.95)	1.0		
	CT	271 (36.52)	990 (47.80)	0.53 (0.41-0.69)	13.64	<0.001

	TT	42 (5.66)	295 (14.24)	0.32 (0.20-0.50)	15.16	<0.001
30	rs3114020 (ABCG2; 88162514; intron)					
	CC	433 (58.04)	793 (38.35)	1.0		
	CT	271 (36.33)	980 (47.39)	0.53 (0.41-0.68)	13.31	<0.001
	TT	42 (5.63)	295 (14.26)	0.32 (0.20-0.50)	15.19	<0.001
31	rs10011796 (ABCG2; 88169725; intron)					
	TT	437 (58.50)	771 (37.30)	1.0		
	CT	266 (35.61)	1002 (48.48)	0.50 (0.39-0.65)	16.29	<0.001
	CC	44 (5.89)	294 (14.22)	0.29 (0.19-0.46)	15.37	<0.001
32	rs4367138 (ABCG2; 88186501; intron)					
	AA	452 (60.51)	963 (46.59)	1.0		
	AG	254 (34.00)	924 (44.70)	0.65 (0.50-0.83)	8.48	<0.001
	GG	41 (5.49)	180 (8.71)	0.60 (0.37-0.98)	4.26	0.039
33	rs55976258 (ABCG2; 88196698; intron)					
	CC	496 (66.40)	1095 (53.00)	1.0		
	CT	233 (31.19)	841 (40.71)	0.71 (0.55-0.92)	7.14	0.009
	TT	18 (2.41)	130 (6.29)	0.40 (0.22-0.75)	5.91	0.004
34	rs11935352 (ABCG2; 88207261; intron)					
	TT	417 (55.82)	803 (38.85)	1.0		
	TC	292 (39.09)	993 (48.04)	0.65 (0.50-0.83)	9.70	0.001
	TT	38 (5.09)	271 (13.11)	0.39 (0.25-0.62)	13.25	<0.001
35	rs13120819 (ABCG2; 88239525;)					
	AA	320 (42.84)	606 (29.32)	1.0		
	AG	352 (47.12)	1017 (49.20)	0.70 (0.54-0.91)	5.31	0.007

	GG	75 (10.04)	444 (21.48)	0.37 (0.25-0.54)	15.72	<0.001
36	rs7656113 (ABCG2; 88245384; upstream)					
	CC	394 (52.82)	815 (39.39)	1.0		
	CA	314 (42.09)	965 (46.64)	0.72 (0.56-0.92)	5.05	0.009
	AA	38 (5.09)	289 (13.97)	0.34 (0.22-0.54)	13.14	<0.001

SLC2A9: Solute carrier family 2, facilitated glucose transporter member 9; MEPE: Matrix, Extracellular, Phosphoglycoprotein; SPP1: Secreted Phosphoprotein 1; PKD2: Polycystin 2; ABCG2: ATP-Binding Cassette, Subfamily G, Member 2; aOR (95% CI): odds ratio and 95% confidence intervals were adjusted by potential confounders: ¥: the p-values were estimated by chi-square test and transformed by negative logarithm. §: the p-values were estimated by logistic regression after adjustment of potential confounders.
