# Supplementary material for: ABCG2 contributes to the development of gout and hyperuricemia in a genome-wide association study
Source: Sci Rep. 2018 Feb 16;8:3137. doi: 10.1038/s41598-018-21425-7 (PMC5816657; doi:10.1038/s41598-018-21425-7)
Supplement: Supplementary file 2 — Supplementary Figure 1 [file 41598_2018_21425_MOESM2_ESM.pdf]

Title: ABCG2 contributes to the development of gout and hyperuricemia in a genome-wide association study

Chung-Jen Chen[1,2], Chia-Chun Tseng[3], Jeng-Hsien Yen[4,5], Jan-Gowth Chang[6], Wen-Cheng Chou[7], Hou-Wei Chu[7], Shun-Jen Chang[8,*], Wei-Ting Liao[9,*]

[1]Division of General Internal Medicine, Department of Internal Medicine, Kaohsiung Medical University Hospital, Kaohsiung, Taiwan.
[2]Department of Internal Medicine, College of Medicine, Kaohsiung Medical University, Kaohsiung, Taiwan.
[3]Department of Internal Medicine, Kaohsiung Municipal Ta-Tung Hospital, Kaohsiung, Kaohsiung Medical University, Kaohsiung, Taiwan.
[4]Division of Rheumatology, Department of Internal Medicine, Kaohsiung Medical University Hospital, Kaohsiung, Taiwan.
[5]Graduate Institute of Medicine, College of Medicine, Kaohsiung Medical University, Kaohsiung, Taiwan.
[6]Department of Laboratory Medicine and Epigenome Research Center, China Medical University Hospital, China Medical University, Taichung, Taiwan.
[7]Institute of Biomedical Sciences, Academia Sinica, Taipei, Taiwan.
[8]Department of Kinesiology, Health and Leisure Studies, National University of Kaohsiung, Kaohsiung, Taiwan.
[9]Department of Biotechnology, College of Life Science, Kaohsiung Medical University, Kaohsiung, Taiwan.

*Corresponding author: Shun-Jen Chang, Department of Kinesiology, Health and Leisure Studies, National University of Kaohsiung, Kaohsiung, Taiwan. No. 700, Kaohsiung University Road, Nanzih District, Kaohsiung city, Taiwan. Phone: +886-7-5916679; Fax: +886-7-5919264; E-mail: changsj1104@gmail.com; or Wei-Ting Liao, Department of Biotechnology, College of Life Science, Kaohsiung Medical University, Kaohsiung, Taiwan. Phone: +886-7-3121101 ext 2791; Fax: +886-7-3125339; E-mail: wtliao@kmu.edu.tw.

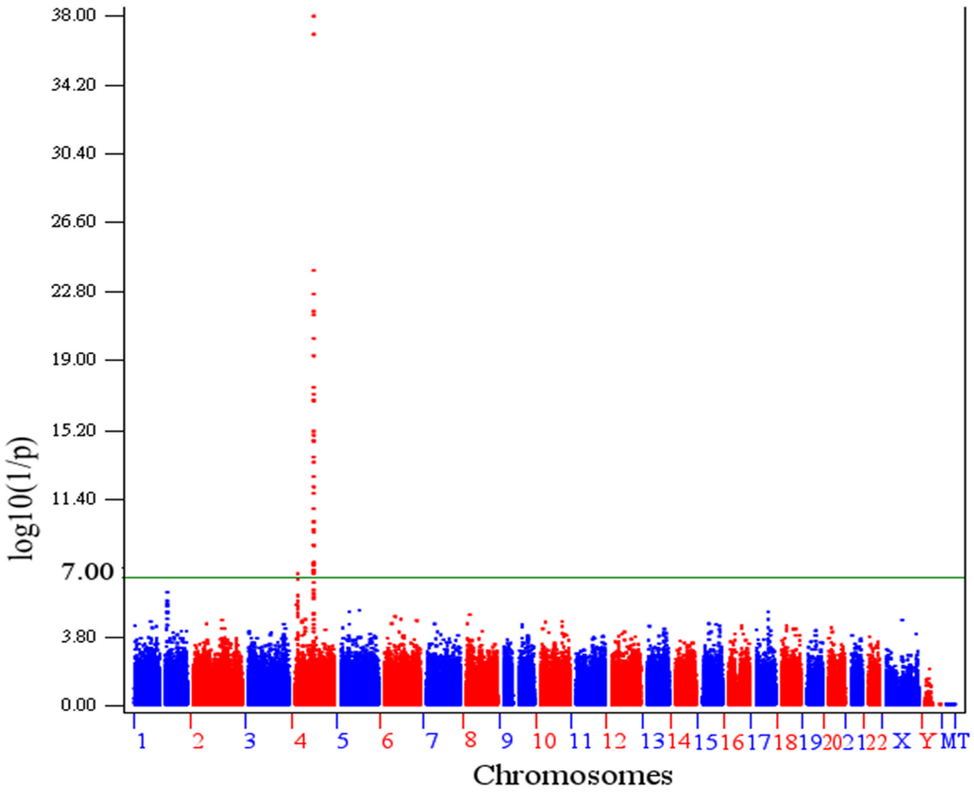

Supplementary figure 1. The Manhattan plot displays the p-values which were estimated by chi-square test from 753 gout patients and 2543 normal controls in a non-matched design. A total of 37 SNPs revealed significant associations with gout disease ($p<10^{-7}$).
