# Supplementary material for: ABCG2 contributes to the development of gout and hyperuricemia in a genome-wide association study
Source: Sci Rep. 2018 Feb 16;8:3137. doi: 10.1038/s41598-018-21425-7 (PMC5816657; doi:10.1038/s41598-018-21425-7)
Supplement: Supplementary file 3 — Supplementary Figure 2 [file 41598_2018_21425_MOESM3_ESM.pdf]

Title: ABCG2 contributes to the development of gout and hyperuricemia in a genome-wide association study

Chung-Jen Chen[1,2], Chia-Chun Tseng[3], Jeng-Hsien Yen[4,5], Jan-Gowth Chang[6], Wen-Cheng Chou[7], Hou-Wei Chu[7], Shun-Jen Chang[8,*], Wei-Ting Liao[9,*]

[1]Division of General Internal Medicine, Department of Internal Medicine, Kaohsiung Medical University Hospital, Kaohsiung, Taiwan.
[2]Department of Internal Medicine, College of Medicine, Kaohsiung Medical University, Kaohsiung, Taiwan.
[3]Department of Internal Medicine, Kaohsiung Municipal Ta-Tung Hospital, Kaohsiung, Kaohsiung Medical University, Kaohsiung, Taiwan.
[4]Division of Rheumatology, Department of Internal Medicine, Kaohsiung Medical University Hospital, Kaohsiung, Taiwan.
[5]Graduate Institute of Medicine, College of Medicine, Kaohsiung Medical University, Kaohsiung, Taiwan.
[6]Department of Laboratory Medicine and Epigenome Research Center, China Medical University Hospital, China Medical University, Taichung, Taiwan.
[7]Institute of Biomedical Sciences, Academia Sinica, Taipei, Taiwan.
[8]Department of Kinesiology, Health and Leisure Studies, National University of Kaohsiung, Kaohsiung, Taiwan.
[9]Department of Biotechnology, College of Life Science, Kaohsiung Medical University, Kaohsiung, Taiwan.

*Corresponding author: Shun-Jen Chang, Department of Kinesiology, Health and Leisure Studies, National University of Kaohsiung, Kaohsiung, Taiwan. No. 700, Kaohsiung University Road, Nanzih District, Kaohsiung city, Taiwan. Phone: +886-7-5916679; Fax: +886-7-5919264; E-mail: changsj1104@gmail.com; or Wei-Ting Liao, Department of Biotechnology, College of Life Science, Kaohsiung Medical University, Kaohsiung, Taiwan. Phone: +886-7-3121101 ext 2791; Fax: +886-7-3125339; E-mail: wtliao@kmu.edu.tw.

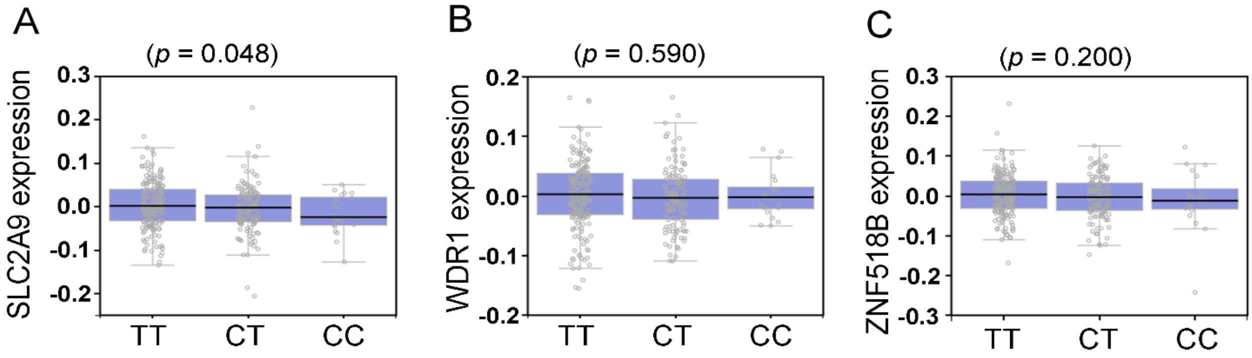

Supplementary Figure 2. The associations between SNP rs9999470 and expression of SLC2A9 (A), WDR1 (B) and ZNF518B (C) genes were estimated by eQTL analysis. The gene expressions of SNP rs9999470 in whole-blood samples were queried from the genotype-tissue expression (GTEx) portal. CLNK gene is not sufficiently expressed and is excluded. The number of samples harboring TT, CT, and CC alleles was 190, 128, and 20, respectively.
